# Simultaneous morphology manipulation and upconversion luminescence enhancement of β-NaYF_4_:Yb^3+^/Er^3+^ microcrystals by simply tuning the KF dosage

**DOI:** 10.1038/srep12745

**Published:** 2015-08-03

**Authors:** Mingye Ding, Daqin Chen, Shilong Yin, Zhenguo Ji, Jiasong Zhong, Yaru Ni, Chunhua Lu, Zhongzi Xu

**Affiliations:** 1 College of Materials & Environmental Engineering, Hangzhou Dianzi University, Hangzhou 310018, P. R. China; 2 State Key Laboratory of Materials-Orient Chemical Engineering, College of Materials Science and Engineering, Nanjing Tech University, Nanjing 210009, P. R. China; 3 College of Chemistry & Materials Engineering, Changshu Institute of Technology, Changshu 215500, Jiangsu, P. R. China

## Abstract

A strategy has been adopted for simultaneous morphology manipulation and upconversion luminescence enhancement of β-NaYF_4_:Yb^3+^/Er^3+^ microcrystals by simply tuning the KF dosage. X-ray power diffraction (XRD), field emission scanning electron microscopy (FE-SEM), transmission electron microscopy (TEM), X-ray photoelectron spectroscopy (XPS) and photoluminescence spectra (PL) were used to characterize the samples. The influence of molar ratio of KF to Y^3+^ on the crystal phase and morphology has been systematically investigated and discussed. It is found that the molar ratio of KF to Y^3+^ can strongly control the morphology of the as-synthesized β-NaYF_4_ samples because of the different capping effect of F^−^ ions on the different crystal faces. The possible formation mechanism has been proposed on the basis of a series of time-dependent experiments. More importantly, the upconversion luminescence of β-NaYF_4_:Yb^3+^/Er^3+^ was greatly enhanced by increasing the molar ratio of KF to RE^3+^ (RE = Y, Yb, Er), which is attributed to the distortion of local crystal field symmetry around lanthanide ions through K^+^ ions doping. This synthetic methodology is expected to provide a new strategy for simultaneous morphology control and remarkable upconversion luminescence enhancement of yttrium fluorides, which may be applicable for other rare earth fluorides.

In recent years, the synthesis of inorganic nano-/microstructures with controllable morphologies and accurately tunable sizes has attracted much attention not only for fundamental scientific interest but also for their potential applications in the fields of photoelectric device, sensor, catalysis, biological labeling, imaging and drug delivery^1^,^2^,^3^,^4^. It is generally accepted that most of the applications of such materials strongly depend on various parameters, including crystal structure, morphology, size, and dimensionality. Subsequently, simultaneous control over shape, size and phase purity of crystals has been becoming the research focus and one of the challenging issues. Until now, a variety of inorganic crystals, such as oxides, oxyfluorides, fluorides, sulfides, hydrates and other compounds, have been prepared with different shapes and sizes by various methods^5^,^6^,^7^,^8^. However, the precisely architectural manipulation of inorganic functional materials with predictable size, shape and crystal phase is still a challenging and urgent task, owing to the complexity of crystal structures and compositions of materials. To clarify these issues clearly, a deep understanding on the nature of shape evolution and phase transition is still needed. As a result, it is very important for us to establish the relationship between the observed complex phenomena of crystal growth with the underlying fundamental theories and principles, which could be regarded as a reference to controllable synthesis of other inorganic materials.

As a significant class of rare earth compounds, rare earth (RE) fluorides have been become a research focus in the material field due to their unique applications in optical communications, three-dimensional displays, solid-state laser, photocatalysis, solar cells, biochemical probes and medical diagnostics^9^,^10^,^11^,^12^,^13^. Among them, NaYF_4_ has been regarded as one of the most excellent host lattices for performing multicolor upconversion (UC) luminescence of the doped RE ions, due to its low phonon energy, high chemical stability and good optical transparency over a wide wavelength range^14^,^15^,^16^,^17^. As we known, the crystal structure of NaYF_4_ exhibits two crystallographic forms, namely, cubic (α-) and hexagonal (β-) phases, depending on the synthesis conditions and methods^18^. Previous studies have indicated that the hexagonal polymorph exhibits considerable enhanced UC emissions compared with the cubic one^14^,^15^. Consequently, how to obtain pure β-NaYF_4_ is crucial in successfully achieving high luminescence performance. Till now, many efforts have been dedicated to exploring excellent routes to the synthesis of hexagonal NaYF_4_ with various sizes and shapes, such as nanospheres, nanoplates, nanorods, nanotubes, microrods, microtubes, microshpheres, micro-bipyramids, microplates and microprisms^19^,^20^,^21^,^22^. However, it is still limited on the investigation of the mechanism underlying the shape and phase evolution of NaYF_4_ microcrystals. A deep understanding on the dynamic process governing nucleation and growth of the complex fluoride microcrystals is further needed.

Compared with other phosphors, such as organic fluorophores and quantum dots, lanthanide ions doped β-NaYF_4_ crystals have many advantages, including sharp emission peaks, large anti-Stokes shifts, long-lived excited electronic states and high photostability^23^,^24^,^25^. But in spite of these advances, improvements are still needed to optimize UC luminescence properties for further potential commercialization. The remarkable challenge for us is how to further enhance the UC intensities of RE ions doped β-NaYF_4_ crystals, which has considerable significance to their applications. So far, several attempts have been devoted to improving UC intensity via internal adjustment and external approaches, such as sensitizing mechanisms^26^, the formation of core-shell structure^27^, the introduction of non-lanthanide ions^28^,^29^ and the incorporation of noble metals^30^,^31^. Among these methods, co-doping with non-lanthanide ions provides an alternative approach to enhance UC luminescence intensity by adjusting the crystal field symmetry.

Herein, we demonstrate a facile and effective hydrothermal process to synthesize β-NaYF_4_ microcrystals using KF as fluoride source. In our experiments, the KF serves two purposes: (1) to tune morphology of the final products based on different capping effect of F^−^ ions on the different crystal faces; (2) to tailor the local crystal field of host lattice by K^+^ ions doping. By simply tuning the molar ratio of KF to Y^3+^, regular β-NaYF_4_ crystals with controllable morphologies can be obtained. In addition, the phase and morphology evolution process as well as the formation mechanism have been systematically investigated and discussed in detail. Meanwhile, significant enhancement of UC luminescence intensity was also observed in β-NaYF_4_:Yb, Er microparticles by simply tuning the KF dosage. To the best of our knowledge, simultaneous morphology control and UC luminescence enhancement for Yb, Er co-doped β-NaYF_4_ microcrystals has been reported for the first time, and KF is rarely used as fluoride source.

## Results and Discussion

### Structures and Morphologies of As-prepared NaYF_4_ samples

**Figure 1:**
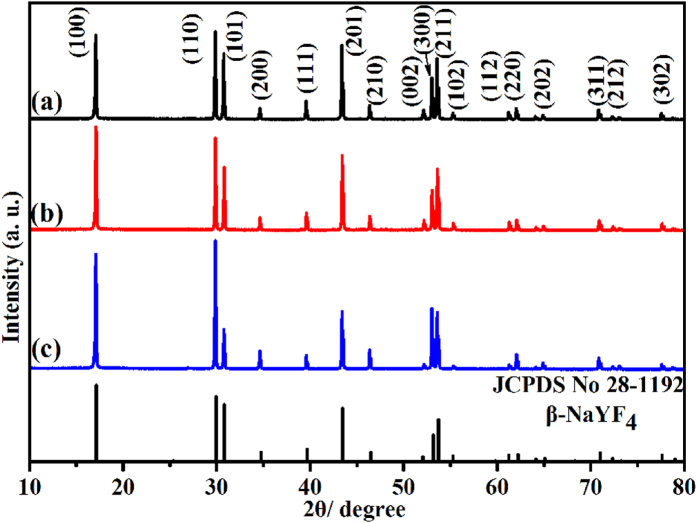
XRD patterns of β-NaYF_4_ samples prepared with different molar ratio of KF to Y^3+^. (**a**) KF/Y^3+^ = 16; (**b**) KF/Y^3+^ = 30; (**c**) KF/Y^3+^ = 50. The standard data of hexagonal NaYF_4_ (JCPDS No. 28-1192) is shown as a reference.

Here, we mainly focus on the effect of the KF/Y^3+^ molar ratios on the phases and morphologies of the final products. In our experiments, the other synthetic parameters were set as reaction temperature 220 °C, reaction time 24 h, Na_3_Cit 1 mmol. Figure 1 shows the XRD patterns of these samples obtained at different molar ratio of KF/Y^3+^ as well as standard data of pure hexagonal NaYF_4_ phase for comparison. As shown, all crystals exhibit diffraction patterns corresponding to the hexagonal phase of NaYF_4_ according to JCPDS No. 28-1192. The crystal structure of the hexagonal phase is determined with lattice parameters of a = 0.596 and c = 0.353 nm, space group P6_3_/mmc^15^. No trace of characteristic peaks is detected for other impurity peaks such as KYF_4_, YF_3_, indicating that the simple hydrothermal method is a feasible route to synthesize pure β-NaYF_4_ microcrystals using KF as fluoride source. Moreover, careful observation reveals that the relative diffraction peak intensity of XRD patterns varies with different molar ratio of KF to Y^3+^, implying the morphology evolution of as-prepared β-NaYF_4_ crystals.

**Figure 2:**
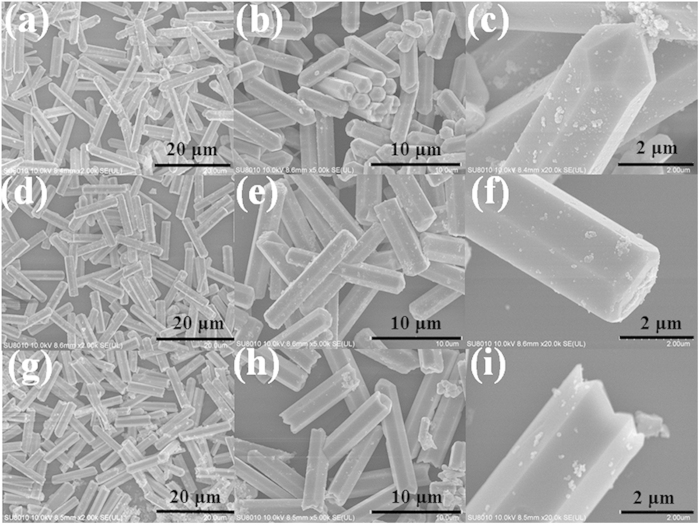
FE-SEM images of β-NaYF_4_ samples prepared with different molar ratio of KF to Y^3+^. (**a**–**c**) KF/Y^3+^ = 16; (**d**–**f**) KF/Y^3+^ = 30; (**g**–**i**) KF/Y^3+^ = 50.

The typical morphology evolution of β-NaYF_4_ microcrystals obtained with increasing KF/Y^3+^ molar ratio is presented in Fig. 2. As shown, the molar ratio of KF to Y^3+^ has a profound influence on the morphologies of the as-synthesized samples. At low molar ratio of KF to Y^3+^ (KF/Y^3+^ = 16), the SEM images in Fig. 2a reveal that the sample is composed of a large quantity of microrods with uniform size of 11.8 μm in length and 2.3 μm in diameter. From the images of a higher magnification (Fig. 2b) and a typical individual microrod (Fig. 2c), we can see that the products are prismatic microrods with smooth and flat surfaces as well as sharp ends. Furthermore, the ends of rods are of hexagonal pyramid structure, as shown in Fig. 2c. At a medium molar ratio of KF to Y^3+^ (KF/Y^3+^ = 30), the general images of β-NaYF_4_ sample are shown in Fig. 2d. It clearly indicates that the as-prepared product consists of a great deal of hexagonal microprisms with prefect uniformity, monodispersity and well-defined crystallographic facets. The mean size of microprism is calculated to be about 2.6 μm in diameter and 12.1 μm in length. Further investigation under higher magnification (Fig. 2e,f) indicates that both tops and bottoms of these microprisms exhibit flat planes. At high molar ratio of KF to Y^3+^ (KF/Y^3+^ = 50), the regular hexagonal prism-shaped β-NaYF_4_ with an average size of 11.5 μm in length and 2.8 μm in diameter are observed from Fig. 2g. A magnified SEM image (Fig. 2h) reveals that the large-scale, regular and monodisperse prismatic microrods with soomth and flat surfaces are obtained in this experimental condition. Interestingly, the surfaces of top/bottom have very regular concave centers, as depicted in Fig. 2i. It’s worth mentioning that the above-mentioned experiments have been repeated three times at least and the same conclusion could be drawn from these experiments. From the above investigations, it can be concluded that the morphology of products can be tuned accordingly with no further change in particle size by changing the molar ratio of KF/Y^3+^.

### Growth Mechanism

**Figure 3:**
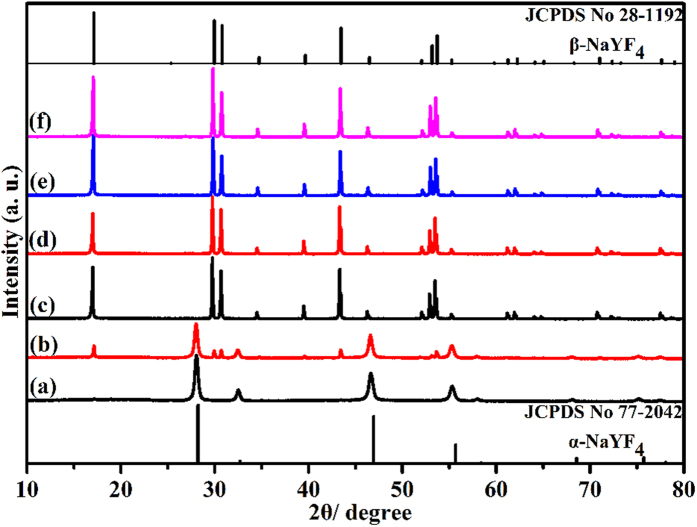
XRD patterns for NaYF_4_ samples (KF/Y^3+^ = 50) as a function of reaction time. (**a**) 1 h, (**b**) 2 h, (**c**) 4 h, (**d**) 8h, (**e**) 12 h, (**f**) 24 h. The standard data of cubic NaYF_4_ (α-NaYF_4_) and hexagonal NaYF_4_ (β-NaYF_4_) are given as a reference.

To understand the formation process of β-NaYF_4_ microcrystals with different morphologies, reaction samples have been carefully investigated by quenching the reaction at different time intervals. Although we know that the reaction doesn’t stop immediately after the autoclave is removed from the heater because of heat transfer, we do believe that the products synthesized at that time represent certain stage in the formation process. Figure 3 shows the XRD patterns of the NaYF_4_ samples synthesized with 50:1 KF/Y^3+^ at different reaction times as well as standard data of α-NaYF_4_ (JCPDS No. 77-2042) and β-NaYF_4_ (JCPDS No. 28-1192) phases for comparison. It reveals that the samples exhibit distinctively different XRD patterns at different reaction times. The sample obtained at t = 1 h is pure cubic NaYF_4_ (Fig. 3a). A new hexagonal NaYF_4_ phase emerges in addition to cubic NaYF_4_ phase with the reaction proceeding for 2 h (Fig. 3b). With the further reaction from 2 h to 4 h, the fraction of β-NaYF_4_ increases dramatically while the amount of α-NaYF_4_ decreases. The result indicates that the phase transformation (α → β) takes place through a dissolution-renucleation process^32^,^33^,^34^. When the reaction time increases from 4 h to 24 h, pure β-NaYF_4_ can be successfully obtained, as shown in Fig. 3c–f. Based on the above analysis, it can be concluded that the crystal evolves from cubic phase to mixed phase and ultimately to hexagonal phase with the prolonged reaction time.

**Figure 4:**
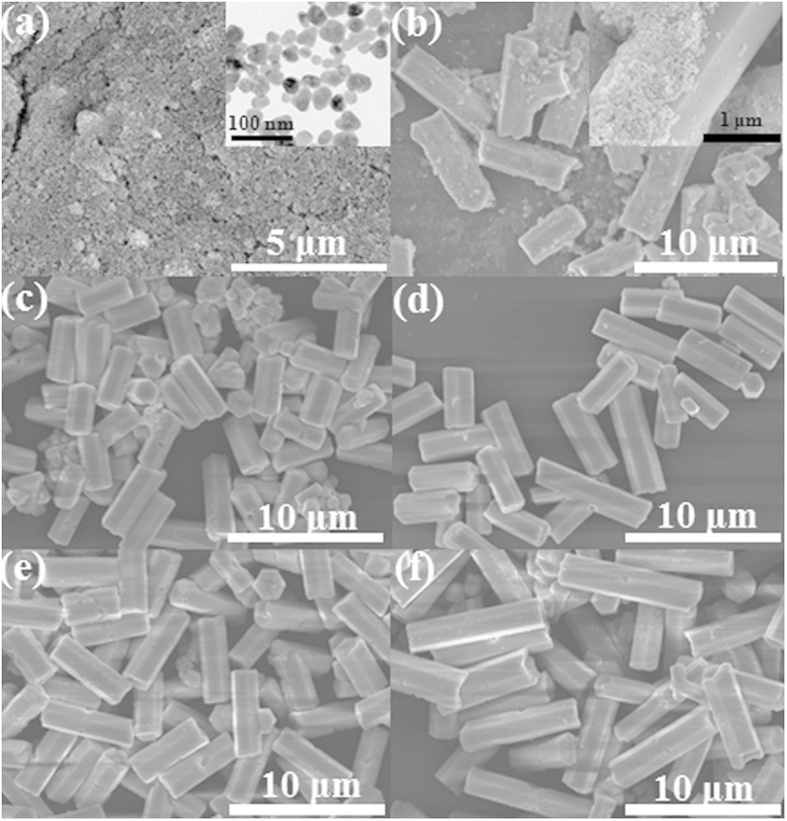
FE-SEM images for NaYF_4_ crystals as a function of reaction time. (**a**) 1 h, (**b**) 2 h, (**c**) 4 h, (**d**) 8 h, (**e**) 12 h, (**f**) 24 h. Inset in Fig. 4(a) is TEM image for the corresponding sample.

At the meantime, the morphologies of the products are carefully investigated by quenching the reaction at different time intervals. Figure 4 shows the corresponding FE-SEM images of the different intermediate samples at different reaction stages. It clearly reveals that the six samples exhibit dramatically different morphologies in the process of crystal growth. At a short reaction time of 1 h, the α-NaYF_4_ sample consists of spherical-like nanoparticles with a mean diameter of 40 nm (Fig. 4a and Fig. S1 in Supplementary Information). But in the present situation, the α-phase of NaYF_4_ is unstable and these nanoparticles would serve as seeds for the growth of β-NaYF_4_ hexagonal microrods by a dissolution-renucleation process. With further reaction, these unstable α-NaYF_4_ nanoparticles convert to β-NaYF_4_ microprisms gradually. After 2 h of growth, the regular and well-defined microprisms begin to appear in the intermediate product. Moreover, a large amount of nanoparticles are attached on the surface of hexagonal microprisms, as shown in Fig. 4b. According to the corresponding XRD pattern, the coexisting of two shapes results from the presence of the mixture crystal phase (α + β). As we known, the cubic NaYF_4_ has isotropic unit cell structure, resulting in an isotropic growth of particles. As a result, spherical-like particles are observed. By comparison, hexagonal NaYF_4_ has anisotropic unit cell structure, which can induce anisotropic growth along crystallographically reactive directions, leading to the formation of hexagonal-shaped structure^35^. On the basis of above analysis, it can inferred that these crystals with different morphologies should be cubic NaYF_4_ (nanoparticles) and hexagonal NaYF_4_ (microprisms), respectively. As reaction time extended to 4 h, the α-NaYF_4_ nanoparticles disappear completely and only the fairly uniform well-defined β-NaYF_4_ microprisms exist. The result also reveals that the phase transition (α → β) can directly induce obvious change in the morphology of NaYF_4_ crystals. As shown in Fig. 4c, the mean length and diameter of rods could be estimated to be 5.2 μm and 1.0 μm, respectively. With the further reaction from 8 h to 24 h, there are no further change in morphology, but the size of microprisms increases from 8.8 μm to 11.6 μm in length and from 1.9 μm to 2.8 μm in diameter (Fig. 4d–f), indicating the longitudinal and transversal growth of NaYF_4_ microprisms along with the reaction time. We also investigate the growth process of β-NaYF_4_ crystals synthesized with other KF/Y^3+^ molar ratios (KF/Y^3+^ = 20, 25, and 40). As can be seen from the XRD patterns (Fig. S2-S4 in Supplementary Information), these crystals exhibit very similar phase transformation process to that of these three samples. In addition, the FE-SEM images (Fig. S5-S7 in Supplementary Information) also reveal a similar phase transformation process (α → β) to these three products.

**Figure 5:**
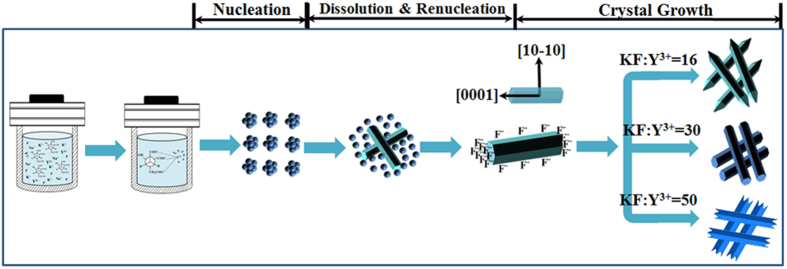
Schematic illustration for the formation of hexagonal NaYF_4_ products with various morphologies under different molar ratio of KF to Y^3+^.

Based on the above results, a possible phase and morphology evolution mechanism is shown in Fig. 5 and described as follows. At the beginning, the citrate anions (Cit^3−^) introduced into the reaction system can form complexes with Y^3+^ ions through strong coordination interaction. At the same time, KF dissolves in the aqueous solution to form K^+^ and F^−^ ions.


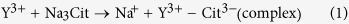



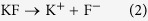


Under the conditions of high temperature and high pressure, the chelating ability of the Y^3+^ − Cit^3−^ complexes would be weakened by slow degrees during hydrothermal process, resulting that the Y^3+^ ions could be released gradually. Then Na^+^, K^+^ and F^−^ ions in this solution react with Y^3+^ ions to generate small nuclei. In a very short reaction time, these nuclei quickly aggregate together and grow into cubic-phased K_x_Na_(1−x)_YF_4_ nanoparticles.


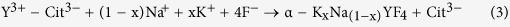


However, these grown up α-K_x_Na_(1−x)_YF_4_ nanoparticles are thermodynamically unstable and evolve inevitably to hexagonal K_x_Na_(1−x)_YF_4_ seeds through dissolution-renucleation process. Meanwhile, this phase transformation (α → β) results in dramatic morphology change of the samples, which could be related to different characteristic unit cell structures for varying crystallographic phases. The dissolution-reconstruction process of cubic-phased nanoparticles preferentially happens at the circumferential edges of each prismy microrod along crystallographically reactive direction, resulting in the formation of rod-like β-K_x_Na_(1−x)_YF_4_ with a well-defined cross-section. With the further reaction, the morphology of products changes from spherical nanoparticles into short hexagonal microrods.


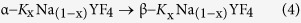


**Figure 6:**
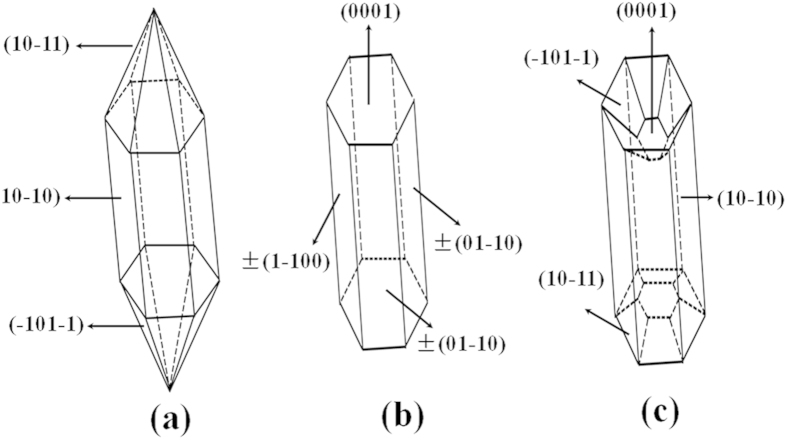
Schematic diagram showing the anisotropy of hexagonal NaYF_4_ microrods synthesized with different KF/Y^3+^ molar ratio. (**a**) 16:1, (**b**) 30:1; (**c**) 50:1.

As we known, the shape evolution of β-NaYF_4_ microcrystals is significantly dependent on external factors such as the molar ratios of KF to Y^3+^ and Na_3_Cit to Y^3+^, the pH values in the solution^36^. In this experiment, the subsequent crystal growth of β-K_x_Na_(1−x)_YF_4_ seeds is significantly affected by the KF/Y^3+^ molar ratio, resulting in different morphologies of hexagonal K_x_Na_(1−x)_YF_4_ microcrystals. Although the exact mechanism is not very clear at present, the explanation for the change of morphology can be provided as follows. According to the general principle of crystal growth, the growth of crystals is related to the relative growth rate of different crystal facets^33^,^37^,^38^. The different growth rate of various crystal planes results in diverse appearance of the crystallite. Generally speaking, crystal facets perpendicular to the fast directions of growth have smaller surface area and show growing faces therefore dominate the morphology of the final crystal. The growth velocity in different crystallographic facets of β-K_x_Na_(1−x)_YF_4_ crystals could be influenced by the coordination effect between F^−^ and Y^3+^ ions. According to the Gibbs-Thompson theory, the relative chemical potential of crystal plane is simply proportional to its surface-atom ratio, determined by the average number of dangling bonds per atom over the entire crystal facet^20^. The capping effect of F^−^ ions could decrease the average number of dangling bonds and further reduce the chemical potential of the crystal plane. Moreover, the different density of Y^3+^ on various crystal planes leads to the difference in chemical potential of crystal facets. Consequently, the chemical potential of different crystal facets could be modified, and the relative growth rates could be affected by different molar ratio of KF to Y^3+^, finally leading to different crystal shapes. For β-K_x_Na_(1−x)_YF_4_ crystals, the density of Y^3+^ on the prismatic planes ({10-10} crystal planes) is bigger than that on the top/bottom facets ({0001} crystal planes), resulting in the selective adsorption ability of F^−^ ions on the prismatic facets being bigger than that on the top/bottom planes. Finally, the relative growth rate along [0001] is much quicker than that of along 10-10, resulting in the hexagonal microrods with long length and high aspect ratio. The difference in the top ends of hexagonal microrods is relate to the different capping effect of F^−^ on the {10–11}, {–101–1} and {0001} crystal planes, as depicted in Fig. 6. At low F^−^ concentration, the capping effect of F^−^ on the {10–11} and {–101–1} crystal planes is greater than it on the {0001} plane. Consequently, the growth rate of {10-11} and {–101–1} crystal facets is faster than that of {0001} planes, resulting in the formation of sharp ends. At medium F^−^ concentration, the capping effect of F^−^ ions on the {0001} facets is greater and the fast growing faces ({0001} crystal planes) therefore induce to the flat ends. The concave structure is observed at the top/bottom facets by further increasing the F^−^ concentration in the solution. The presence of the concave ends demonstrates that the growth rate of the prismatic side facets ({10-10} planes) is a little faster than that of the top/bottom facets ({0001} planes).

### Upconversion luminescence properties

**Figure 7:**
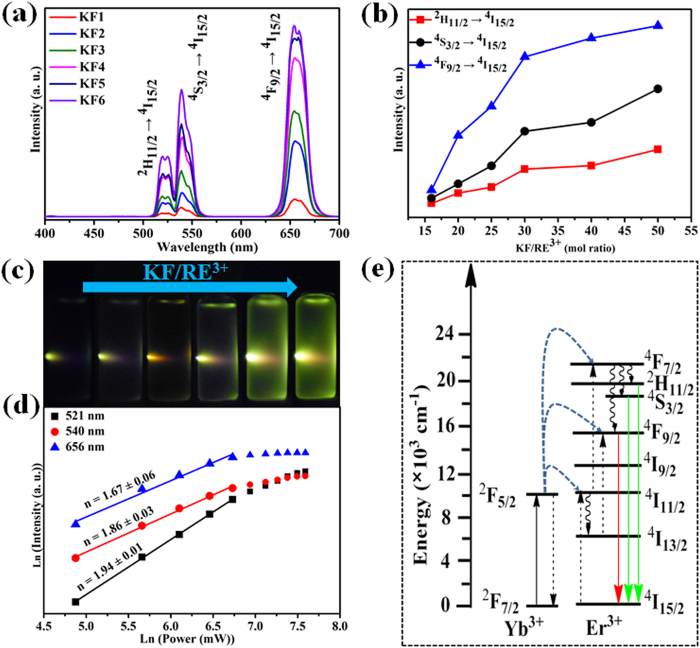
(**a**) Room-temperature UC emission spectra of β-NaYF_4_:20%Yb^3+^, 2%Er^3+^ samples synthesized with different molar ratio of KF to RE^3+^ (Excitation: 980 nm; Power density: ~0.25 W/mm^2^); (**b**) integrated emission intensity comparison of the green region (^2^H_11/2_, ^4^S_3/2_ → ^4^I_15/2_) and red region (^4^F_9/2_ → ^4^I_15/2_) as a function of molar ratio of KF to RE^3+^; (**c**) luminescence photographs of the samples dispersed in ethanol solution under 980 nm laser diode excitation; (**d**) pump power dependence of the UC emissions in β-NaYF_4_:20%Yb^3+^, 2%Er^3+^; (**e**) proposed energy transfer mechanism in the Yb^3+^, Er^3+^ co-doped β-NaYF_4_ crystals.

To investigate the UC luminescence properties of β-NaYF_4_ synthesized with different molar ratios of KF to RE^3+^, Yb^3+^/Er^3+^ are selected as co-doped ion pairs to form β-NaYF:Yb^3+^, Er^3+^ microcrystals. Figure 7 shows the UC emission spectra of β-NaYF_4_:20%Yb^3+^, 2%Er^3+^ samples (20 mg powder is dispersed in 10 mL ethanol under ultrasound treatment) synthesized by different molar ratio of KF to RE^3+^ under 980 nm laser diode excitation (power density: 0.2 W/mm^2^). As can be seen clearly, the six samples show the same emission peaks yet with quite different emission intensity. As shown in Fig. 7a, the characteristic UC emission bands centered at 521 nm, 540 nm and 656 nm can be ascribed to ^2^H_11/2_ → ^4^I_15/2_ (green), ^4^S_3/2_ → ^4^I_15/2_ (green) and ^4^F_9/2_ → ^4^I_15/2_ (red) transitions of Er^3+^, respectively^39^. It is worth noticing that the UC luminescence intensities in both the green and red regions increase notably with the increment of molar ratio of KF to RE^3+^. Figure 7b exhibits the integral intensity of green and red emissions as a function of the KF dosage. By increasing the molar ratio of KF to RE^3+^, the integral intensities of 521 nm, 540 nm and 656 nm emissions are enhanced dramatically. The integrated green (500–600 nm) and red (600–700 nm) emissions in KF6 sample are measured to be about 15 and 12 times as high as that of KF1 sample (Table S1 in Supporting Information). In addition, the similar trend of green and red emissions also suggests the same upconversion pathways for them. To visualize the enhancement of UC emission, the corresponding luminescence photographs of β-NaYF_4_:20%Yb^3+^, 2%Er^3+^ crystals synthesized with different KF dosage are provided in Fig. 7c. The green emission of six samples can be seen clearly by naked eyes. Moreover, the emission intensity of KF6 sample is the strongest, which agrees with the results in Fig. 7a,b. To accurately demonstrate enhancement of UC luminescence, the quantum yields of the KF1 and KF6 samples were measured. Importantly, the UC efficiencies of KF1 and KF6 are roughly estimated to be ~0.3% and ~1.6%, respectively. The detailed measuring procedure for quantum yield has been presented in supporting information (Fig. S8). Additionally, the obvious UC enhancement can also observed in Yb^3+^/Tm^3+^ co-doped β-NaYF_4_ samples (Fig. S9 in Supplementary Information). Notably, the integrated blue emission of the β-NaYF_4_:20%Yb^3+^, 0.5%Tm^3+^ sample obtained with KF/RE^3+^ molar ratio 50 are enhanced by about 80 times (Table S2 in Supporting information), resulting in the strongest blue emission under the excitation of 980 nm laser diode. The results are quite similar to the Yb^3+^/Er^3+^ co-doped β-NaYF_4_ samples, revealing the generality of the approach. In order to deeply investigate the relevant UC mechanism in the as-synthesized β-NaYF_4_:20%Yb^3+^, 2%Er^3+^ crystals, the excitation power-dependent UC emissions of green and red are calculated accordingly. It is generally known that the output UC emission intensity (*I*_*uc*_) is proportional to the infrared excitation power (*I*_*IR*_): *I*_*uc*_ ∝ *(I*_*IR*_)^*n*^, where *n* is the absorbed photon numbers per visible photon emitted, and its values can be acquired from the slope of the fitted line of the plot of log(*I*_*uc*_) versus log(*I*_*IR*_)^40^,^41^. The pump power dependence of the UC emissions in KF6 sample under 980 nm LD excitation is presented in Fig. 7d. As shown, the slopes of the linear fit of log(*I*_*uc*_) versus log(*I*_*IR*_) for 521 nm, 540 nm and 656 nm are 1.67, 1.86 and 1.94, respectively. The result indicates that only two-photon process is involved to produce the green and red UC emissions, whereas a saturation effect can be observed at relatively higher excitation power. Based on the above results, the proposed UC mechanism in β-NaYF_4_:Yb^3+^/Er^3+^ under 980 nm LD excitation is shown in Fig. 7e and briefly described as follows^42^. Firstly, the electron of Yb^3+^ is excited from ^2^F_7_ to ^2^F_5_ level in β-NaYF_4_: Yb^3+^/Er^3+^ microcrystals under 980 nm LD excitation. An initial energy transferred from Yb^3+^ ions in the ^2^F_5/2_ state to Er^3+^ ions populates the ^4^I_11/2_ level of Er^3+^ ions. Then, a second 980 nm photon transferred by the adjacent Yb^3+^ ions can populate the ^4^F_7/2_ level of Er^3+^ ions, whose energy lies in the visible region. The Er^3+^ ions can relax nonradiatively to the level of ^2^H_11/2_, ^4^S_3/2_ and ^4^F_9/2_. Through a two-photon UC process, the dominant green and red emissions are observed by these transitions from the aforementioned states to ^4^I_15/2_ level.

**Figure 8:**
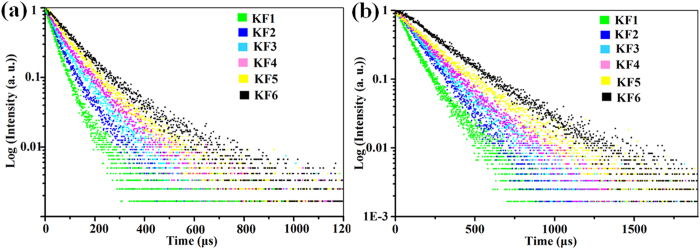
The decay curves for the ^4^S_3/2_ → ^4^I_15/2_ (**a**) and ^4^F_9/2_ → ^4^I_15/2_ (**b**) emissions of Er^3+^ in β-NaYF_4_:20%Yb^3+^, 2%Er^3+^ samples prepared with different molar ratio of KF to RE^3+^.

In order to demonstrate the UC enhancement more theoretically, the emission decay curves of ^4^S_3/2_ → ^4^I_15/2_ (540 nm) and ^4^F_9/2_ → ^4^I_15/2_ (656 nm) transitions in the six samples were measured at the excitation wavelength of 980 nm, as shown in Fig. 8. The effective experimental lifetime is evaluated using


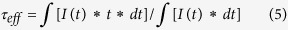


where *I(t)* represents the luminescence intensity at time t after the cutoff of the excitation light^43^. It can be seen clearly that the lifetimes of ^4^S_3/2_ and ^4^F_9/2_ states in β-NaYF_4_:Yb^3+^/Er^3+^ samples are prolonged gradually with the increase of molar ratio of KF to RE^3+^. Furthermore, the variation trend of lifetimes is also consistent with the enhancement of UC luminescence intensity. The average lifetimes of ^4^S_3/2_ and ^4^F_9/2_ states of Er^3+^ ions for β-NaYF_4_:Yb^3+^/Er^3+^ crystals synthesized with different KF/RE^3+^ molar ratio are listed in Table 1 (Fig. S10 and Fig. S11 in Supplementary Information). Obviously, the longest lifetimes of ^4^S_3/2_ and ^4^F_9/2_ states are measured to be 58.71 μs and 276.69 μs, which is found in KF6 sample prepared with 50:1 molar ratio of KF:RE^3+^. As we known, the experimental lifetime of an excited state, *τ*, is determined by the theoretical lifetime (*τ*_*rad*_), the nonradiative transition rate (*W*_*NR*_) and the energy transfer rate (*W*_*ET*_), which could be expressed as^44^


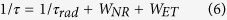


According to the UC mechanism and experimental results, it can be concluded that the energy transfer process between Yb^3+^ and Er^3+^ hasn’t been changed by tuning the KF dosage. Moreover, the nonradiative transition rate should not have obvious effect on the great enhancement of UC luminescence because of the same experimental and excitation conditions for these samples. Therefore, we can ascribe the as-observed much longer lifetimes (*τ*) to the increase of theoretical lifetimes (*τ*_*rad*_), namely the modification of lattice parameters. The introduction of K^+^ ions can tailor the local crystal field of the host lattice, and therefore can modify the theoretical lifetimes of Er^3+^ ions through slightly changing their wave functions.

### Enhancement mechanism for UC emissions

**Figure 9:**
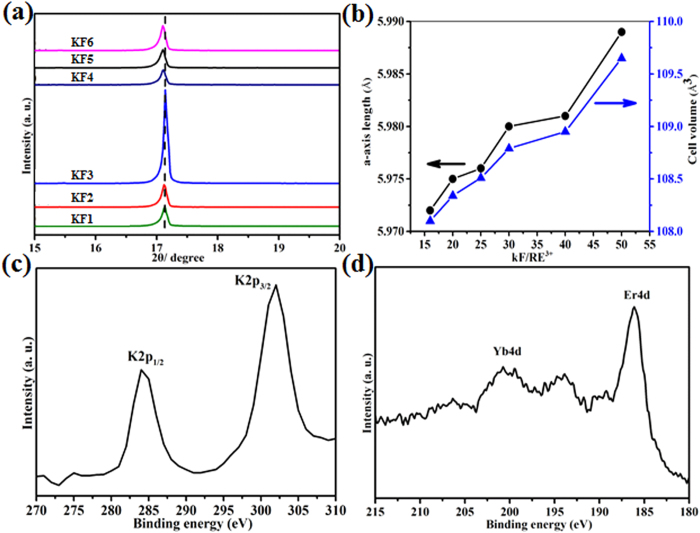
(**a**) The main diffraction peaks, (**b**) Variation of cell volume and a-axis of β-NaYF_4_:Yb, Er microcrystals synthesized with different molar ratio of KF to RE^3+^; High resolution XPS spectra of K 2p (**c**) Yb and Er 4d (**d**) in KF6 sample.

What accounts for the obvious enhancement of UC luminescence when tuning the KF dosage in our experiments? Three important factors for UC enhancement should be in consideration: crystal phase, particle size and morphology, local crystal field. First of all we should investigate the crystal phase, particle size and morphology of β-NaYF_4_:Yb^3+^/Er^3+^ crystals synthesized with the addition of different KF dosage. The XRD patterns and FE-SEM images of as-prepared β-NaYF_4_:Yb^3+^/Er^3+^ samples are presented in Fig. S12 and Fig. S13, respectively. It is evident that all the peaks can be indexed to pure hexagonal-phased NaYF_4_ according to JCPDS card (No. 28-1192). No additional peaks can be detected, indicating that the addition of KF has not lead to the formation of other impurity phases. Although the morphology of crystals has changed a lot, the specific surface areas of the six samples show a little distinction from each other (Table S3 in Supplementary Information). Moreover, the luminescent property of phosphors is relate to their specific surface area of the materials. Consequently, it is obvious that the first two factors should not have an obvious effect on marked enhancement of UC luminescence. However, careful observation reveals that the position of the main diffraction peak at 17.1° in the XRD pattern shifts slightly towards small angles as the molar ratio of KF to RE^3+^ increases (Fig. 9a). The variation of lattice parameters and unit cell volume with the KF dosage were calculated form the observed XRD data and are presented in Fig. 9b. According to the Bragg’s law (*nλ* = *2dsinθ*), the decrease of Bragg angle (*θ*) indicates that the spacing between the planes in atomic lattice (*d*) increases, resulting in the expansion of unit cell. Considering that KF is used as fluorine source and r_K_ (1.51–1.57 Å) > r_Na_ (1.13–1.53 Å), we can easily infer that some K^+^ ions are doped in β-NaYF_4_ host lattice and may occupy lattice sites by the substitution of Na^+^ ions. Furthermore, X-ray photoelectron spectroscopy (XPS) was used to determine the successful incorporation of rare earth ions (Yb^3+^, Er^3+^) and K^+^ ions into the β-NaYF_4_ host matrix. As shown in Fig. 9c, the peaks observed at 285.5 and 302.0 eV can be assigned to the binding energy of K2p_1/2_ and K2p_3/2_, respectively^45^. The characteristic peaks at 198.8 and 186.5 eV can be assigned to the binding energy of Yb 4d and Er 4d, respectively (Fig. 9d)^46^. The EDS and ICP-AES analyses (Table 2 and Fig. S15 in Supplementary Information) are also used to confirm the successful incorporation of K^+^, Yb^3+^, Er^3+^ ions into β-NaYF_4_ matrix. Evidently, with increasing of the KF dosage in the product, K content increases gradually, and the Yb and Er contents keep unchanged. Therefore, it is believed that the introduction of K^+^ ions into the host lattice leads to the UC enhancement.

**Figure 10:**
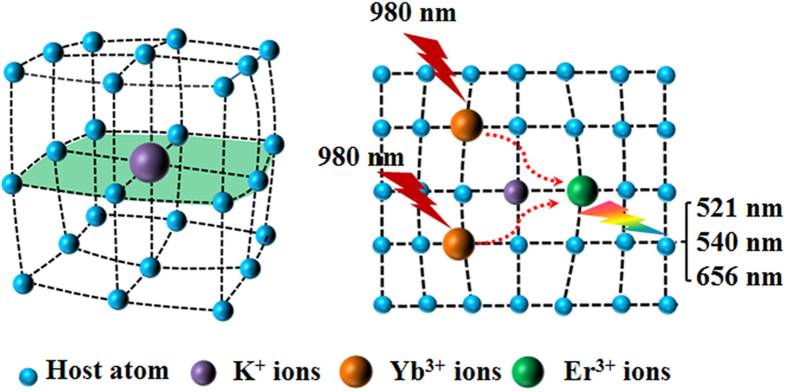
The possible change in the β- NaYF_4_ crystal lattice after K^+^ ions doping.

Based on the above results, we can provide the following explanation for this obvious enhancement of UC luminescence. According to the single-crystal X-ray powder diffraction data, the crystal structure of hexagonal NaYF_4_ with the P6_3_/mmc has three types of cation sites in a unit cell: one for rare earth ions (site 1a), one for both rare earth and sodium ions (site 1f), and the third for sodium ions (site 2h). Site 1a and 1f both have C_3h_ symmetry, whereas site 2h has C_s_ symmetry^18^,^47^. When trivalent lanthanide ions (Yb^3+^, Er^3+^) are doped into β-NaYF_4_ lattice by isomorphic substitution of Y^3+^ ions without charge compensation, only one kind of substitutional site with a crystallographic site symmetry of C_3h_ could be observed in the unit cell. It is well known that the electric-dipole transitions between 4f^n^ configuration (f-f) with the same parity are parity-forbidden for free lanthanide ions according to the quantum selection rules. However, such prohibition can be broken by mixing of opposite-parity configuration, resulting that the electric-dipole transitions can be weakly allowed in crystal lattice. In order to greatly increase the electric diploe transitions probability, an asymmetric crystal field is required. When K^+^ ions are doped in crystal lattice, in view of the bigger ionic radius of K^+^ relative to that of Na^+^, K^+^ ions only occupy the lattice sites by the substitution of Na^+^ ions in the crystal, corresponding to the situation presented in Fig. 10 48. As a result, the coordination shell around site 1f originally statistically distributed by Y^3+^ and Na^+^ ions could be perturbed seriously. Accordingly, the displacement patterns of various Y/Na coordination shell around each subset of lanthanide ion can be slightly different. According to the microscopic model of disorder, the local site symmetry around the lanthanide ions may descend from C_3h_ to lower symmetries C_s_. For rare earth ions embedded in solid materials, lower crystal symmetry caused by tailoring the crystal structure is generally favorable for higher UC emission intensity. Therefore, the introduction of K^+^ ions into β-NaYF_4_ crystal lattice would change the crystal field and lower the local crystal field symmetry around lanthanide ions, resulting in the enhancement of UC luminescence intensity^49^. Tailoring the local crystal field of host lattice has become a general explanation for increasing UC emission intensity when introducing non-luminescent ions, such as Li^+^, Bi^3+^, Sc^3+^, Fe^3+^ ions^29^,^46^,^50^,^51^. However, the direct evidence of tailoring local crystal field is still lacking. So, investigation on the local structure and site symmetry of lanthanide ions, for example, by using high-resolution photoluminescence spectroscopy at low temperature (10 K), are further needed to provide such proof.

In conclusion, we have described a facile and general strategy for simultaneous morphology manipulation and UC luminescence enhancement of β-NaYF_4_:Yb^3+^/Er^3+^ samples using KF as fluoride source. Through the simple manipulation of the KF/Y^3+^ molar ratio, regular β-NaYF_4_ microcrystals with predictable shapes have been synthesized. A mechanism for how the molar ratio of KF to Y^3+^ influences the anisotropic growth and morphology evolution of β-NaYF_4_ crystals is proposed. Based on the phase and morphology evolution, the possible formation mechanism for hexagonal NaYF_4_ is discussed in detail. Notably, the UC luminescence intensity of β-NaYF_4_:Yb^3+^/Er^3+^ sample is significantly enhanced by increasing the KF dosage. It is found that the doping of K^+^ ions into β-NaYF_4_ crystal lattice can tailor the local crystal field and lower the local crystal field symmetry around lanthanide ions, which is the main reason for the UC enhancement. This study provides a reference for simultaneous morphology control and UC luminescence enhancement of rare earth fluorides, which will have great potential in fields of sensors, solar cells and photocatalysis.

## Methods

### Chemicals

All of the chemical reagents used in this experiment are analytical grade and were received without further purification. Deionized water was used throughout. YCl_3_.6H_2_O (99.99%), YbCl_3_.6H_2_O (99.99%) and ErCl_3_.6H_2_O (99.9%) were purchased from Beijing Founde Star Science & Technology Co., Ltd (China). KF.2H_2_O (99%), C_6_H_5_Na_3_O_7_.2H_2_O (99%) were provided by Sinopharm Chemical Reagent Co., Ltd (China).

### Preparation

β-NaYF_4_:Yb^3+^, Er^3+^ microcrystals have been synthesized via a facile hydrothermal method assisted with trisodium citrate. In a typical procedure for the synthesis of β-NaYF_4_: 20%Yb^3+^, 2% Er^3+^, 1.56 mmol YCl_3_.6H_2_O, 0.40 mmol YbCl_3_.6H_2_O, 0.04 mmol ErCl_3_.6H_2_O were firstly dissolved in 10 mL H_2_O with magnetic stirring to form rare earth chloride solution. Then this solution was added into 20 mL aqueous solution containing 2 mmol trisodium citrate (Na_3_Cit) to form the metal-citrate complex. After vigorous stirring for 30 min, 30 mL aqueous solution containing different amounts (32, 40, 50, 60, 80, 100 mmol) of KF.2H_2_O was introduced into the above solution. After additional agitation for 15 min, the as-obtained mixing solution (60 mL) was transferred into a Teflon bottle (100 mL) held in a stainless steel autoclave, sealed, and maintained at 220 °C for 24 h. As the autoclave was cooled to room temperature naturally, the precipitates were separated by centrifugation, washed with deionized water and ethanol in sequence, and then dired in air at 80 °C for 12 h. In addition, different molar ratios (16:1, 20:1, 25:1, 30:1, 40:1, 50:1) of KF to RE^3+^ (RE = Y, Yb, Er) and hydrothermal treatment times (0.5 h, 1 h, 3 h, 8 h, 12 h, 24 h) were selected to investigate the effects of these factors on the morphological, structural and luminescent properties of the as-obtained sampleaccs. According to the KF/RE^3+^ molar ratio, the resulted samples were denoted as KF1, KF2, KF3, KF4, KF5, and KF6, resspectively. These as-syntheszied products were used to characterize without any further purification.

### Characterization

Powder X-ray diffraction (XRD) measurements were performed on a ARL X’ TRA diffractometer at a scanning rate of 10°/min in the 2θ range from 10° to 80° with Cu Kα radiation (λ = 0.15406 nm). SEM micrographs were obtained using a field emission scanning electron transmission microscope (FE-SEM, SU8010, Hitachi). Transmission electron microscopy (TEM) was recorded on a JEM-200CX with a field emission gun operating at 200 kV. Images were acquired digitally on a Gatan multiple CCD camera. The chemical compositions were determined by inductively coupled plasma (ICP) technique using a Perkin-Elmer Optima 3300DV spectrometer. X-ray photoelectron spectroscopy (XPS) spectra were performed with a PHI5000 VersaProbe system, using a monochromatic Al Kα X-ray source. UC emission spectra were recorded with a Jobinyvon FL3-221 fluorescence spectrophotometer equipped with a 980 nm LD (laser diode) Module (K98SA3M-54W, China) as the excitation source. Upconversion decay lifetimes were measured with a customized UV to mid-infrared steady-state and phosphorescence lifetime spectrometer (FSP920-C, Edinburgh) equipped with a digital oscilloscope (TDS3052B, Tektronix) and a tunable mid-band OPO pulse laser as excitation source (410–2400 nm, 10 Hz, pulse width ≤ 5 ns, Vibrant 355II, OPOTEK). All the measurements were performed at room temperature.

## Additional Information

**How to cite this article**: Ding, M. *et al.* Simultaneous morphology manipulation and upconversion luminescence enhancement of β-NaYF_4_:Yb^3+^/Er^3+^ microcrystals by simply tuning the KF dosage. *Sci. Rep.* **5**, 12745; doi: 10.1038/srep12745 (2015).

## Supplementary Material

Supplementary Information

## Figures and Tables

**Table 1:** Lifetimes of the ^4^S_3/2_ and ^4^F_9/2_ states of Er^3+^ ions for β-NaYF_4_:20%Yb^3+^, 2%Er^3+^ samples prepared with different molar ratio of KF to RE^3+^.

Sample	KF:RE^3+^ (molar ratio)	τ_1_/μs (^4^S_3/2_)	τ_2_/μs (^4^F_9/2_)
KF1	16	23.97	121.54
KF2	20	38.22	171.93
KF3	25	38.54	179.14
KF4	30	46.14	215.20
KF5	40	47.47	216.69
KF6	50	58.71	276.69

**Table 2:** EDS and ICP-AES analysis results of β-NaYF_4_:Yb^3+^/Er^3+^ samples prepared with different molar ratio of KF to RE^3+^.

Sample	Na:K:Y:Yb:Er	
	EDS result	ICP-AES result
KF1	0.992:0.008:0.791:0.190:0.019	0.994:0.006:0.781:0.199:0.020
KF2	0.981:0.019:0.786:0.196:0.018	0.977:0.023:0.795:0.188:0.017
KF3	0.957:0.043:0.771:0.208:0.021	0.962:0.038:0.778:0.202:0.020
KF4	0.932:0.068:0.789:0.191:0.020	0.925:0.075:0.780:0.197:0.023
KF5	0.907:0.093:0.768:0.213:0.019	0.913:0.087:0.796:0.189:0.015
KF6	0.879:0.121:0.781:0.197:0.022	0.891:0.109:0.779:0.205:0.016
