# Supplementary material for: Simultaneous morphology manipulation and upconversion luminescence enhancement of β-NaYF4:Yb3+/Er3+ microcrystals by simply tuning the KF dosage
Source: Sci Rep. 2015 Aug 3;5:12745. doi: 10.1038/srep12745 (PMC4522666; doi:10.1038/srep12745)
Supplement: Supplementary Information [file srep12745-s1.pdf]

Supporting information

Simultaneous morphology manipulation and upconversion luminescence enhancement of β-$NaYF_4$:Yb^{3+}/Er^{3+} microcrystals by simply tuning the KF dosage

Mingye Ding[1,2], Daqin Chen[1,*], Shilong Yin[3], Zhenguo Ji[1], Jiasong Zhong[1], Yaru Ni[2], Chunhua Lu[2,*], Zhongzi Xu[2]

[1] College of Materials & Environmental Engineering, Hangzhou Dianzi University, Hangzhou 310018, P. R. China, [2] State Key Laboratory of Materials-Orient Chemical Engineering, College of Materials Science and Engineering, Nanjing Tech University, Nanjing 210009, P. R. China, [3] College of Chemistry & Materials Engineering, Changshu Institute of Technology, Changshu 215500, Jiangsu, P. R. China

E-mail address: dqchen@hdu.edu.cn (D. Q. Chen), chhlu@njtech.edu.cn (C. H. Lu)

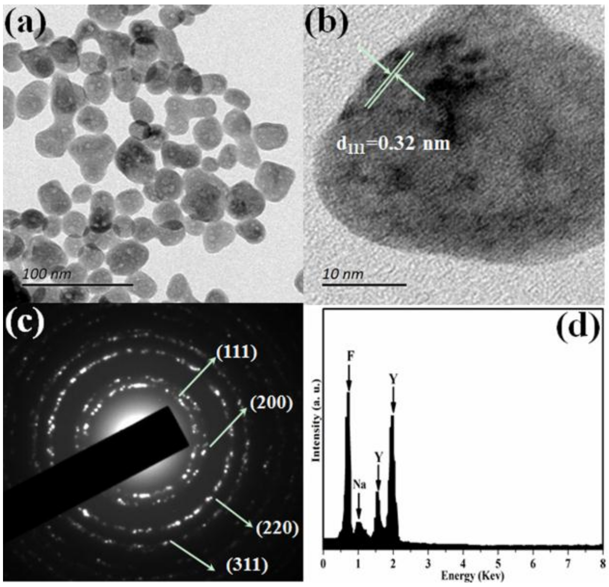

Fig. S1 (a) TEM image, (b) HRTEM image, (c) SAED, and (d) EDS of the sample obtained at 1 h.

To provide a further insight into the as-obtained nanoparticles, TEM investigation and EDS analysis are also performed. The TEM image and HRTEM micrograph in Fig S1(a) and Fig. S1(b) reveal that the sample consists of uniform and well-dispersed nanoparticles with a diameter about 40 nm. The determined interplanar distances of 0.32 nm between the adjacent lattice planes agrees well with the d_{111} spacing of cubic $NaYF_4$ (JCPDS No. 77-2042). Moreover, the SAED pattern in Fig. S1(c) shows spotty polycrystalline diffraction rings, which can be indexed to the (111), (200), (220), and (311) planes of cubic $NaYF_4$ lattice. Additionally, the EDS in Fig. S1(d) shows a characteristic intensity profile of Na, Y, and F elements.

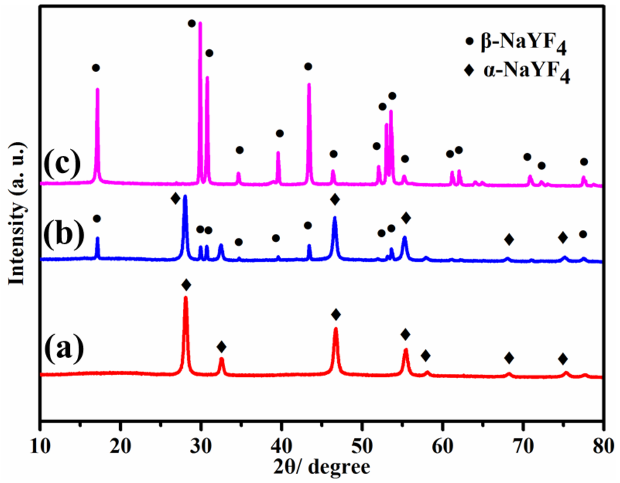

Fig. S2 XRD patterns for hexagonal $NaYF_4$ samples (KF/Y^{3+} = 20) as a function of reaction time. (a) 1 h, (b) 4 h, (c) 12 h.

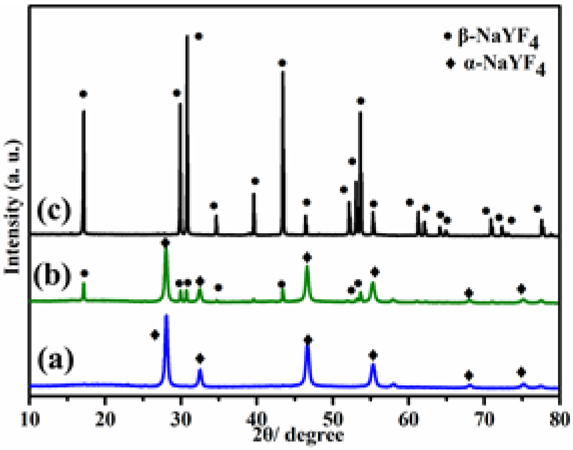

Fig. S3 XRD patterns for hexagonal $NaYF_4$ samples (KF/Y^{3+} = 25) as a function of reaction time. (a) 1 h, (b) 4 h, (c) 12 h.

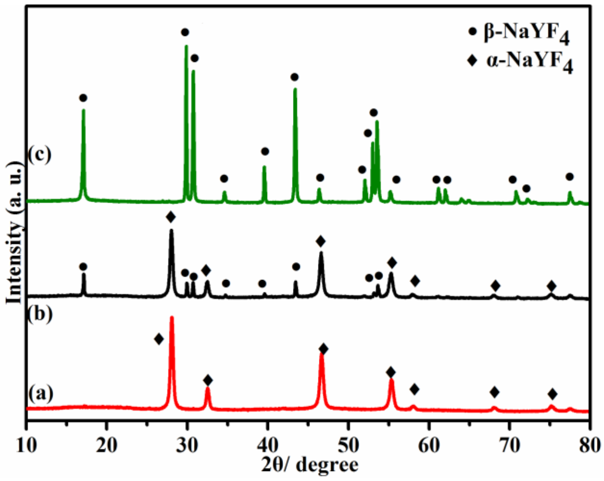

Fig. S4 XRD patterns for hexagonal $NaYF_4$ samples (KF/Y^{3+} = 40) as a function of reaction time. (a) 1 h, (b) 4 h, (c) 12 h.

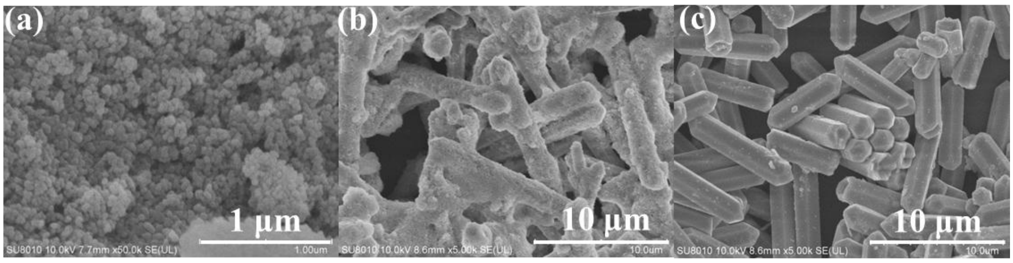

Fig. S5 SEM images for hexagonal $NaYF_4$ samples (KF/Y^{3+} = 20) as a function of reaction time. (a) 1 h, (b) 4 h, (c) 12 h.

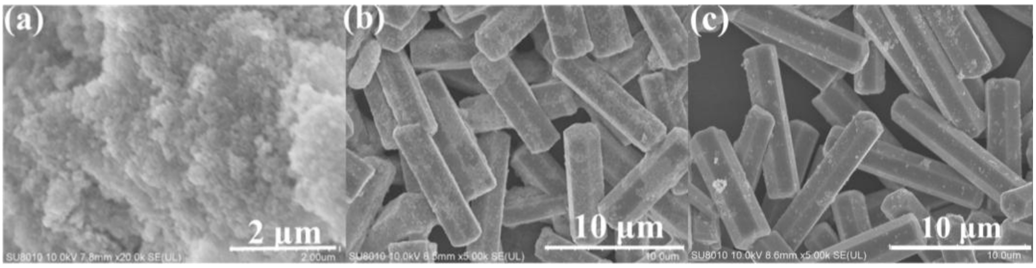

Fig. S6 SEM images for hexagonal $NaYF_4$ samples (KF/Y^{3+} = 25) as a function of reaction time. (a) 1 h, (b) 4 h, (c) 12 h.

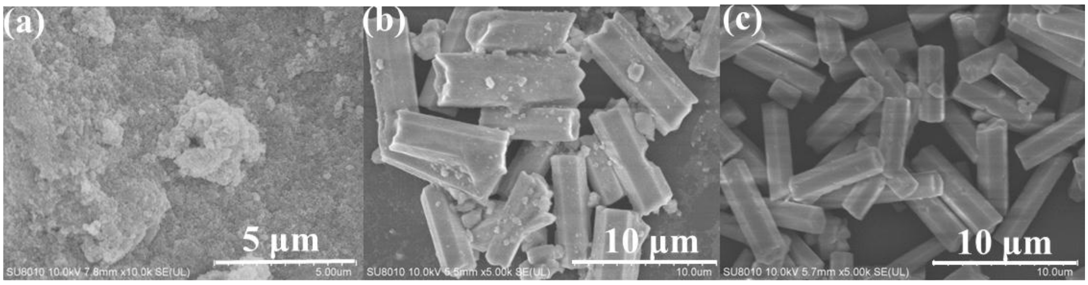

Fig. S7 SEM images for hexagonal $NaYF_4$ samples (KF/Y^{3+} = 40) as a function of reaction time. (a) 1 h, (b) 4 h, (c) 12 h.

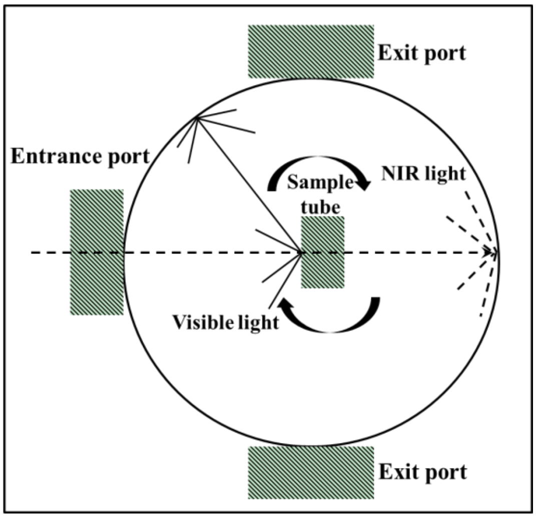

Fig. S8 Experimental setup schematic for measuring quantum yield of upconversion luminescence materials[3].

Quantum yield (QY), defined as the ratio of the emitted photons to the absorbed photons, was

measured by fluorescence spectrophotometer (FLS 980). An integrating sphere was mounted on the spectrofluoremeter with the entrance and exit ports located in 90 ^{o}C geometry. The as-synthesized sample was located in the center of the integrating sphere. All the recorded spectroscopic data were corrected from the spectral responses of both the spectrofluoremeter and the integrating sphere. Based on this setup (Fig. S9), QY is determined sing the following equation[1-3]:

$$\eta = \frac{\text{number of photons emitted}}{\text{number of photons absorbed}} = \frac{L_{sample}}{E_{reference} - E_{sample}}$$

Where η represents QY, L_{sample} is the emission intensity, $E_{reference}$ and E_{sample} are the intensities of the excitation light not absorbed by the reference and the sample, respectively. The undoped reference ($NaYF_4$) was used as the standard reference. The difference in integrated areas between the sample and the reference represents the number of the absorbed photons. The photons emitted were determined by integrating the area of the emission band.

Table S1 The UC luminescence intensity of β-$NaYF_4$:20%Yb^{3+}, 2%Er^{3+} samples synthesized with different molar ratio of KF to RE^{3+}.

**Sample**	**I_{green} (500-600 nm)**	**I_{red} (600-700 nm)**	**Times**	**Times**
**KF1**	15055797	29461835	-	-
**KF2**	46314940	133396100	3.08	4.53
**KF3**	79778810	187525555	5.30	6.37
**KF4**	149397860	302204565	9.92	10.26
**KF5**	168157530	354268720	11.17	12.02
**KF6**	235316530	371779700	15.63	12.62

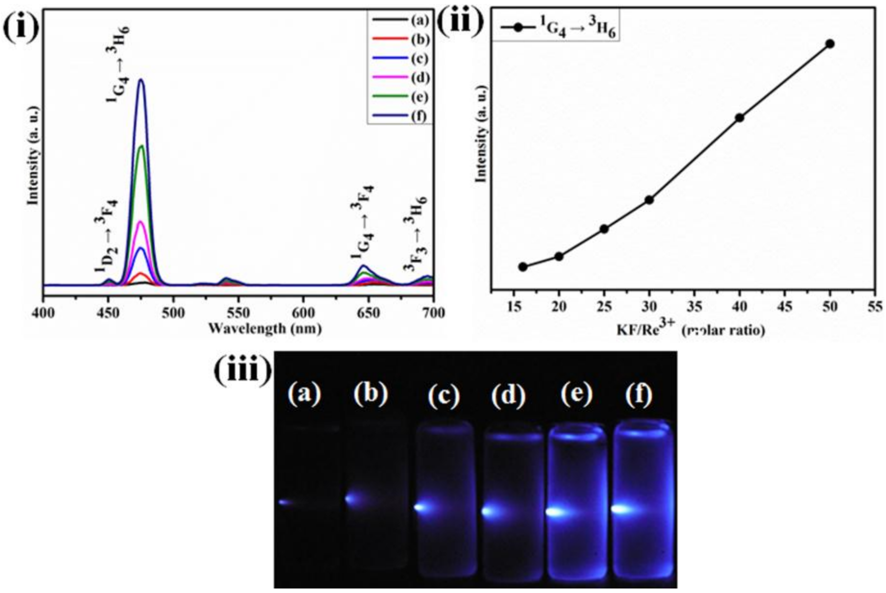

Fig. S9 Room-temperature UC emission spectra (i), integrated emission intensity (ii), and the corresponding luminescence photographs (iii) of β-$NaYF_4$:20%Yb^{3+}, 0.5%Tm^{3+} samples synthesized with different molar ratio of KF to RE^{3+}: (a) KF/RE^{3+} = 16, (b) KF/RE^{3+} = 20, (c) KF/RE^{3+} = 25, (d) KF/RE^{3+} = 30, (e) KF/RE^{3+} = 40, and (f) KF/RE^{3+} = 50.

Table S2 The UC luminescence intensity of β-$NaYF_4$:20%Yb^{3+}, 0.5%Tm^{3+} samples synthesized with different molar ratio of KF to RE^{3+}.

**Sample**	**KF/RE^{3+} (molar ratio)**	**I_{blue} (400-500 nm)**	**Times**
**KF1**	**16**	13930	-
**KF2**	**20**	65460	4.69
**KF3**	**25**	201890	14.49
**KF4**	**30**	345610	24.81
**KF5**	**40**	753420	54.09
**KF6**	**50**	1119670	80.38

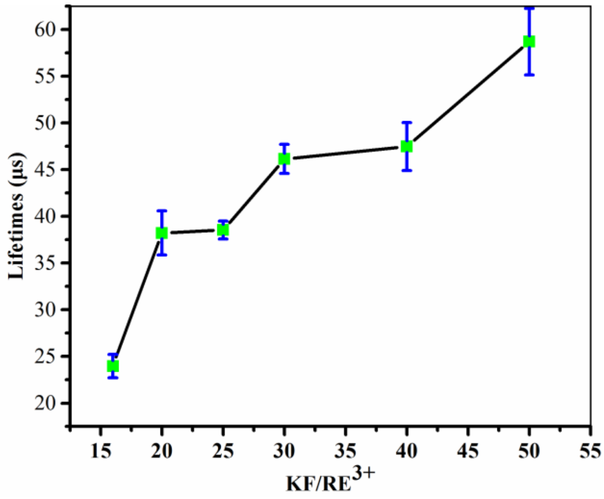

Fig. S10 Lifetimes of the $^4S_{3/2}$ state of Er^{3+} ions for β-$NaYF_4$: 20%Yb^{3+}, 2%Er^{3+} samples synthesized with different molar ratio of KF to RE^{3+}.

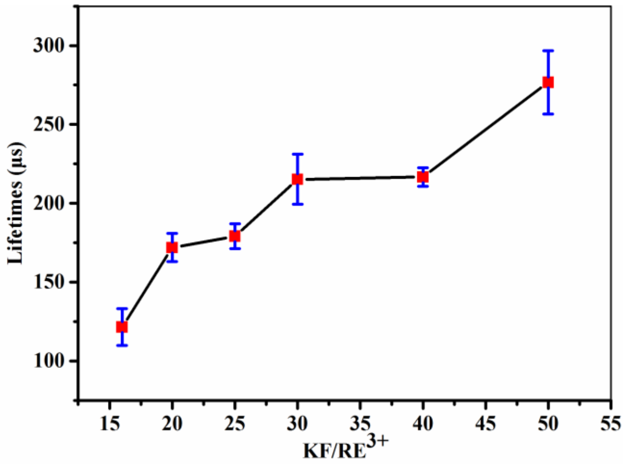

Fig. S11 Lifetimes of the $^4F_{9/2}$ state of Er^{3+} ions for β-$NaYF_4$: 20%Yb^{3+}, 2%Er^{3+} samples synthesized with different molar ratio of KF to RE^{3+}.

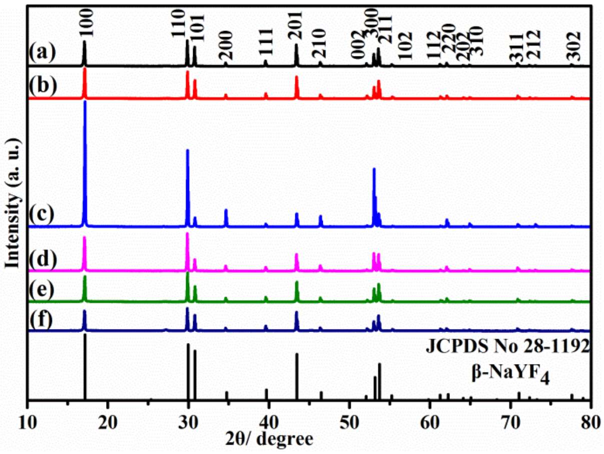

Fig. S12 XRD patterns of β-$NaYF_4$:Yb^{3+}/Er^{3+} samples prepared with different molar ratio of KF to RE^{3+}: (a) KF/RE^{3+} = 16; (b) KF/RE^{3+} = 20; (c) KF/RE^{3+} = 25, (d) KF/RE^{3+} = 30, (e) KF/RE^{3+} = 40, (f) KF/RE^{3+} = 50. The standard data of hexagonal $NaYF_4$ (JCPDS No. 28-1192) is shown as a reference.

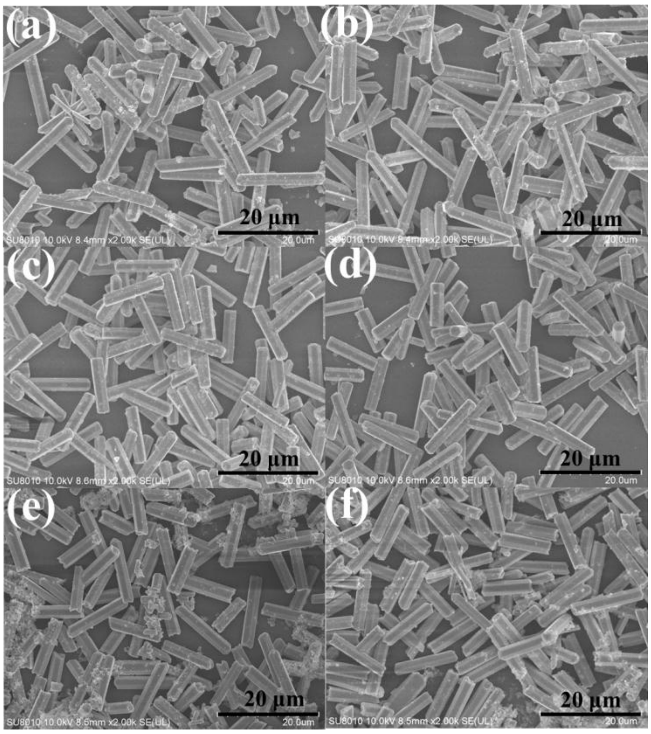

Fig. S13 FE-SEM images of β-$NaYF_4$:Yb^{3+}/Er^{3+} samples synthesized with different molar ratio of KF to RE^{3+}: (a) KF/RE^{3+} = 16; (b) KF/RE^{3+} = 20; (c) KF/RE^{3+} = 25, (d) KF/RE^{3+} = 30, (e) KF/RE^{3+} = 40, (f) KF/RE^{3+} = 50.

Table S3 Summary of the experimental conditions and the corresponding morphologies, dimensions, and specific surface areas of the as-synthesized samples.[a]

Sample	KF:RE^{3+} (molar ratio)	morphology	Diameter (D, μm)	Length (L, μm)	Aspect ratio (L/D)	Specific area (S/V, μm^{-1})
**KF1**	16	Prismatic microrod (conical ends)	2.31	11.8	5.01	1.15
**KF2**	20	Prismatic microrod (conical ends)	2.48	12.38	4.99	1.09
**KF3**	25	Prismatic microrod (flat surfaces)	2.36	11.26	4.77	1.14
**KF4**	30	Prismatic microrod (flat surfaces)	2.66	12.10	4.55	1.04
**KF5**	40	Prismatic microrod (Concave centers)	2.46	11.62	4.72	1.11
**KF6**	50	Prismatic microrod (Concave centers)	2.60	11.58	4.45	1.01

[a] Particle sizes were counted over 180 particles from FE-SEM images. Errors of counting D and L for KF1, KF2, KF3, KF4, KF5, KF6 samples are 7.6%, 8.6%, 5.8%, 8.1%, 9.2%, 6.3%, 8.9%, 9.2%, 9.5%, 10.2%, 7.3%, 9.7%, respectively.

In order to calculate the specific surface area of the as-obtained samples, we start from the simplest case: Prismatic microrods of hexagonal $NaYF_4$, as shown in Fig. S13.

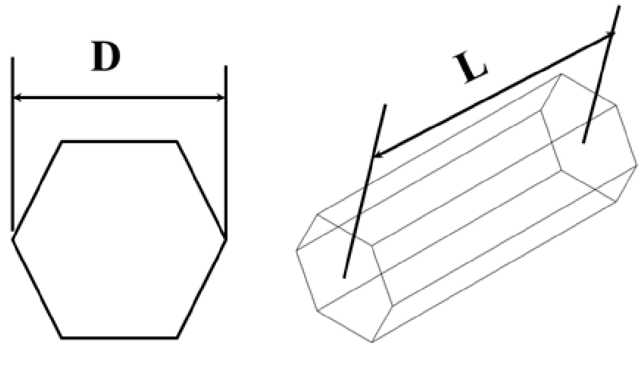

Fig. S14 Schematic diagram showing the anisotropy of the β-$NaYF_4$ hexagonal rod with prismatic structure.

The volume of a prismatic microrod (V) can be described as:

$$V = \frac{3\sqrt{3}D^2L}{4} \quad (1)$$

The surface area of a prismatic microrod (S) is given as follows:

$$S = \frac{3\sqrt{3}D^2}{2} + 3DL \quad (2)$$

Where D is the diameter of a microrod, L is the length of a microrod.

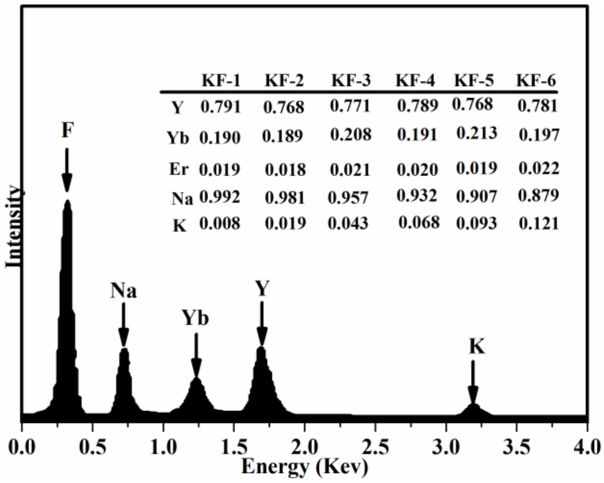

Fig. S15 EDS spectra of β-$NaYF_4$:Yb^{3+}/Er^{3+} samples synthesized with different molar ratio of KF to RE^{3+}.

References

1. Page, R. H. *et al.* Upconversion-pumped luminescence efficiency of rare-earth-doped hosts sensitized with trivalent ytterbium. *J Opt Soc Am B* **15**, 996-1008 (1998).

2. Boyer, J. C. & van Veggel, F. C. J. M. Absolute quantum yield measurements of colloidal $NaYF_4$: Er^{3+}, Yb^{3+} upconverting nanoparticles. *Nanoscale* **2**, 1417-1419 (2010).

3. Dyck, N. C., van Veggel, F. C. J. M. & Demopoulos, G. P. Size-Dependent Maximization of Upconversion Efficiency of Citrate-Stabilized β-phase NaYF4:Yb^{3+},Er^{3+} Crystals via Annealing. *ACS Appl Mater Inter* **5**, 11661-11667 (2013).
